# Effect of lysyl oxidase (LOX) on corpus cavernous fibrosis caused by ischaemic priapism

**DOI:** 10.1111/jcmm.13411

**Published:** 2017-12-26

**Authors:** Liang Gao, Changjing Wu, Fudong Fu, Xuanhe You, Xue Ma, Feng Qin, Tao Li, Run Wang, Jiuhong Yuan

**Affiliations:** ^1^ The Andrology Laboratory West China Hospital Sichuan University Chengdu Sichuan China; ^2^ Department of Urology West China Hospital Sichuan University Chengdu Sichuan China; ^3^ Department of Urology The Second Affiliated Hospital of Chongqing Medical University Chongqing China; ^4^ Department of Pediatric Surgery West China Hospital Sichuan University Chengdu Sichuan China; ^5^ Department of Urology University of Texas M.D. Anderson Cancer Center Houston TX USA

**Keywords:** ischaemic priapism, lysyl oxidase, erectile dysfunction, fibrosis

## Abstract

Penile fibrosis caused by ischemic priapism (IP) adversely affects patients’ erectile function. We explored the role of lysyl oxidase (LOX) in rat and human penes after ischemic priapism (IP) to verify the effects of anti‐LOX in relieving penile fibrosis and preventing erectile dysfunction caused by IP in rats. Seventy‐two rats were randomly divided into six groups: control group, control + β‐aminopropionitrile (BAPN) group, 9 hrs group, 9 hrs + BAPN group, 24 hrs group, and 24 hrs + BAPN group. β‐aminopropionitrile (BAPN), a specific inhibitor of LOX, was administered in the drinking water. At 1 week and 4 weeks, half of the rats in each group were randomly selected for the experiment. Compared to the control group, the erectile function of IP rats was significantly decreased while the expression of LOX in the corpus cavernosum was significantly up‐regulated in both 9 and 24 hrs group. Proliferated fibroblasts, decreased corpus cavernosum smooth muscle cells/collagen ratios, destroyed endothelial continuity, deposited abnormal collagen and disorganized fibers were observed in IP rats. The relative content of collage I and III was not obviously different among the groups. β‐aminopropionitrile (BAPN) could effectively improve the structure and erectile function of the penis, and enhance recovery. The data in this study suggests that LOX may play an important role in the fibrosis of corpus cavernosum after IP and anti‐LOX may be a novel target for patients suffering with IP.

## Introduction

Priapism is a rare urological emergency, which is defined as abnormally prolonged erection for more than 4 hrs in the absence of or after sexual stimulation has ended 1. It can be classified into subtypes of non‐ischaemic (high‐flow), ischaemic (low‐flow) and stuttering (recurrent) 2. The pathophysiology of IP is very complex. At the beginning, obstruction of the penile venous drainage is triggered by all kinds of aetiologies, which lead to local congestion and follows with prolonged erection. If the penis cannot be detumesced in 24 hrs, necrosis or transformation to fibroblast‐like cells of the corpora cavernosum smooth muscle cells (CCSMC) will take place, which will lead to further penile fibrosis and patients’ erectile dysfunction (ED) 3. It has been reported that more than 90% patients are attacked by permanent ED because of IP lasting more than 24 hrs 4.

Lysyl oxidase (LOX) is an extracellular and copper‐dependent monoamine oxidase, which has been proven to work through catalysing the crosslink of lysine residues in collagen I and III, promotes deposition of insoluble elastic fibres and following fibrosis. Our goal was to explore the effects of LOX on penile fibrosis and ED caused by IP using a rat model.

## Materials and methods

Firstly, six Sprague‐Dawley (SD) rats at the ages of 1, 2, 3 and 12 months were respectively killed and their penes were obtained. Further detection was carried out to explore the expression of LOX in penes with age.

Then, seventy‐two adult SD rats (weight: 330–350 g) were randomly divided into six groups: control (vacuum + normal saline [NS]) group, control + β‐aminopropionitrile (BAPN) (vacuum + BAPN) group, 9 hrs group (IP for 9 hrs + NS), 9 hrs + BAPN group, 24 hrs group (IP for 24 hrs + NS) and 24 hrs + BAPN group. BAPN, a specific inhibitor of LOX, was fed immediately after modelling using its fumarate with a dose of 182 mg/kg per day in drinking water (equivalent to 100 mg/kg BAPN free base) 5.

To induce IP, a vacuum device with a pressure of about ‐200 mmHg was used for artificial penile erection. Then, the base of penis was banded by an elastic band with inner diameter of 2 mm and thickness of 1 mm to prevent detumescence. The elastic band was removed after 9 or 24 hrs, which stood for short or long IP duration, respectively (Fig. S1) 6. After 1 week or 4 weeks, six of twelve rats in each group were randomly experimented.

Intracavernous pressure/mean arterial pressure (ICP/MAP) was used to evaluate the erectile function 7. After ICP measurement, the middle section of the penis was harvested. The expression of LOX was detected by Western blot and immunohistochemistry (IHC). Masson trichrome and sirius red staining were used to evaluate the changes of CCSMC/collagen and collagen I/III. Transmission electron microscopy was used to analyse the microchanges in endothelium and collagen.

## Results

Western blot and IHC of corpus cavernosum revealed that the expression of LOX gradually decreased with age (Fig. S2A and C). After the value of LOX/GAPDH was calculated, significances could be found in the 12‐month group compared to 1‐ and 2‐month groups (Fig. S2B).

**Figure 1:**
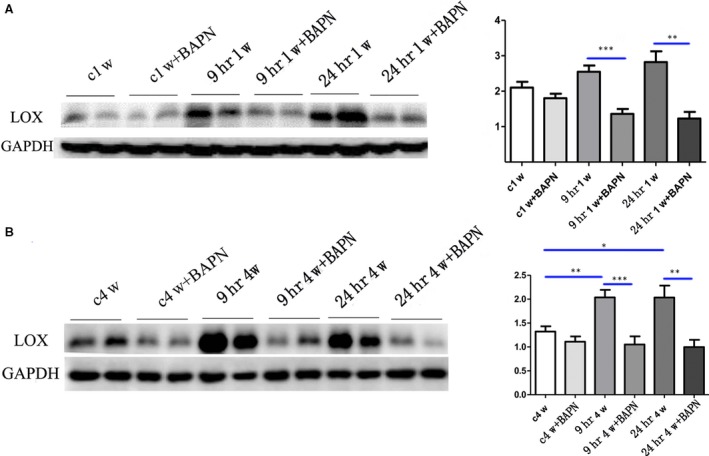
Penile LOX expressions in different groups after 1 (**A**) or 4 (**B**) weeks of ischaemic priapism (IP). c1w: control group for 1 week; 9hr1w: 1 week after IP for 9 hrs; 24hr1w: 1 week after IP for 24 hrs; c4w: control group for 4 weeks; 9hr4w: 4 weeks after IP for 9 hrs; 24hr4w: 4 weeks after IP for 24 hrs; BAPN: β‐aminopropionitrile.

One week after IP, penile level of LOX was up‐regulated both in 9 hrs and 24 hrs groups compared to the control group, but the differences were insignificant. BAPN could significantly down‐regulate the expression of LOX (Fig. 1A). Moreover, LOX in 9 hrs and 24 hrs groups was significantly higher than the control group after 4 weeks. Similar results could be found for the effects of BAPN on LOX expression (Fig. 1B).

Erectile function of rats was significantly damaged after IP no matter whether in 9 hrs group or in 24 hrs group compared to the control group after 1 week. BAPN could significantly improved erectile function (Fig. S3A). Similarly, improved function through use of BAPN could be also demonstrated in the IP rats after 4 weeks (Fig. S3B).

**Figure 2:**
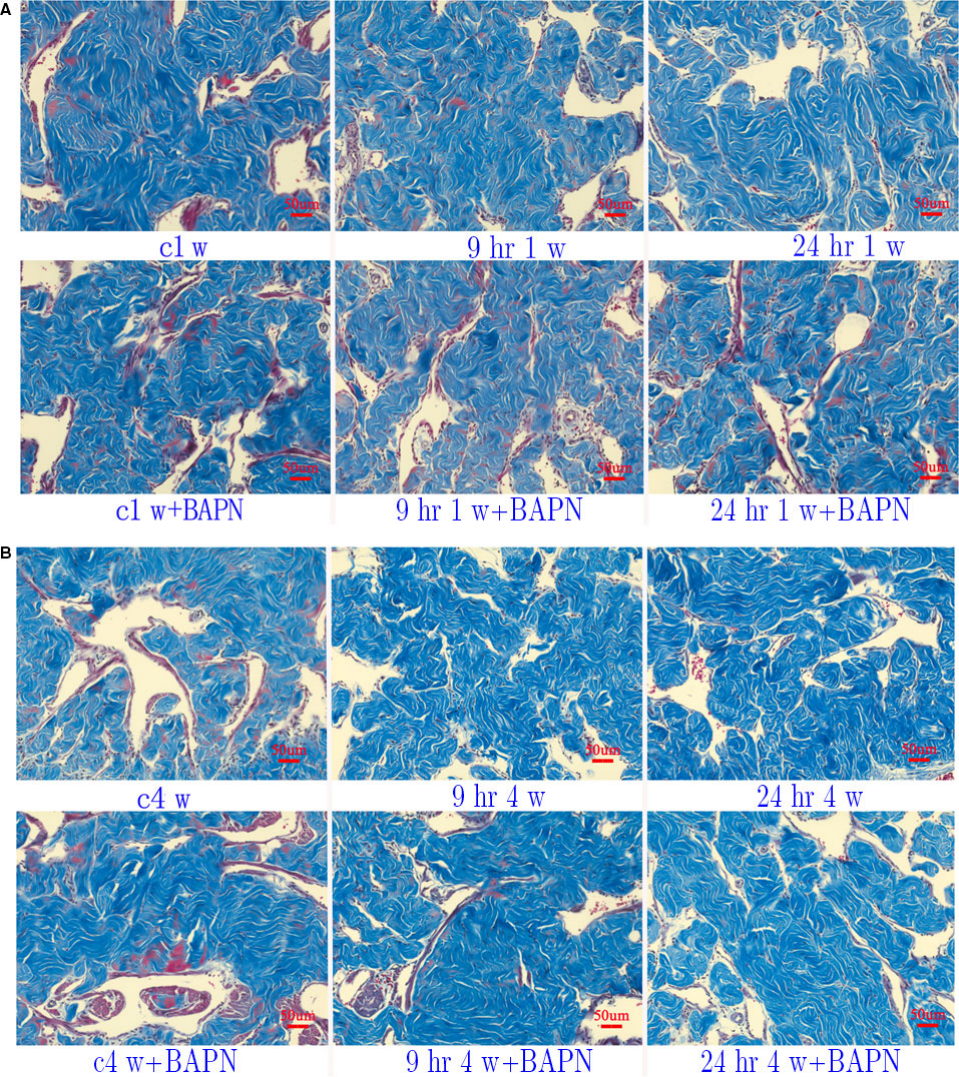
Masson staining (20×) of corpus cavernosum at stages of 1 (**A**) and 4 (**B**) weeks.

We used Masson trichrome to evaluate the content of collagen and CCSMC. Significant decreases in CCSMC/collagen ratio were revealed in 9 hrs and 24 hrs groups compared to the control group at two stages, which provided important evidence of penile fibrosis after IP. Moreover, BAPN could elevate the content of CCSMC to a large degree (Fig. 2). However, no differences or tendencies could be found in the values of collagen I/III by sirius red staining (Fig. S4).

The morphology of endothelium and collagen in the corpus cavernosum was analysed by transmission electron microscopy. Cellular oedema and structural distortion were noticed in the 9 hrs group as well as at the 24 hrs group at the 1 week stage, and the consecutiveness of endothelium in these two groups was severely damaged. The cavernous sinus was filled with many fibroblasts and abnormal collagen. At the 4‐week stage, abnormal collagen deposition, fibroblastic proliferation and tissue oedema were distinctly recovered to a large degree. However, endothelial discontinuity or desquamation was still common (Fig. S5). In 9 hrs and 24 hrs group at stage of 1 week, collagen deposit and fibroblastic proliferation were somewhat decreased by BAPN treatment (Fig. S5). We further analysed changes focusing on collagen, which revealed distortion at 1 week and loose fibrotic bundles at 4 weeks. It was partially recovered after BAPN treatment in two groups at both stages (Fig. S6).

## Discussion

It has been proven that the expression of LOX was significantly decreased in multiple organs with age 8, 9, 10, which implies important roles it might play in the development or maturation of these organs. Our study was the first to reveal that LOX expression in the corpus cavernosum followed the same trend. To avoid the influence of different ages on LOX expression in the corpus cavernosum, strict controls of 1 week and 4 weeks were applied in our study.

In our study, we were also first to explore the role of LOX in IP, which revealed that LOX might promote penile fibrosis after IP. And, treatment against LOX could significantly ameliorate penile fibrosis and improve erectile function at the same time.

Bastos *et al*. carried out an analysis focusing on the composition of penis in a 28‐week‐old foetus, which found that collagen accounted for 47% of normal corpus cavernosum in humans 11. Although this value could elevate to 68% and 73% in Peyronie's disease and IP, the differences were insignificant. 12

Conversely, Costa *et al*. found significantly more collagen and less CCSMC in the corpus cavernosum of IP patients compared to those of normal males. Similarly, others demonstrated significant lower CCSMC/collagen in rats after IP 13, 14. Damaged endothelial continuity could be also found 14.

Current studies almost reached the conclusion that decreased CCSMC/collagen and damaged endothelial structure could happen in IP 15. This would be also an important reason for ED occurring after IP. However, the constituents of collagen in the corpus cavernosum still need further validation.

In summary, LOX may play an important role in IP rats and promote the fibrotic process of the corpus cavernosum after IP. Further study is needed to explore the mechanism of LOX in penile development and whether LOX can be a potential target for IP treatment.

## Author contributions

L.G. designed the study, performed the experiments, including collection of materials, ICP/MAP measurement, data compilation, and drafted the manuscript; C.W., F.F., X.Y. and F.Q. performed Western blot and stainings; X.M. and T.L. performed collection of materials; and R.W. and J.Y. designed the study and wrote the manuscript.

## Conflict of interest

The authors confirm that there is no conflict of interests.

## Supporting information


**Fig. S1** The establishment of ischemic priapism (IP) model.
**Fig. S2** Western Blot (A) and immunohistochemistry (IHC) (C) of LOX in penis from rats with ages of 1, 2, 3 and 12 months, respectively. Statistical analysis (B) revealed significant decreases in 12 months group compared to 1 and 2 months group. LOX: lysyl oxidase.
**Fig. S3** ICP graphs and ICP/MAP for rats in different groups and at stages of 1 (A) and 4 (B) weeks after IP.
**Fig. S4** Sirius red staining (20×) of corpus cavernosum by polarizing microscopy at stages of 1 (A) and 4 (B) weeks.
**Fig. S5** Transmission electron microscopy of corpus cavernosum (12,000×) at stages of 1 (A) and 4 (B) weeks.
**Fig. S6** Transmission electron microscopy of collagen in corpus cavernosum (15,000×) at stages of 1 (A) and 4 (B) weeks.
