# Supplementary material for: Effect of lysyl oxidase (LOX) on corpus cavernous fibrosis caused by ischaemic priapism
Source: J Cell Mol Med. 2017 Dec 26;22(3):2018–22. doi: 10.1111/jcmm.13411 (PMC5824375; doi:10.1111/jcmm.13411)
Supplement: Supplementary file 1 — Fig. S1 The establishment of ischemic priapism (IP) model. Fig. S2 Western Blot (A) and immunohistochemistry (IHC) (C) of LOX in penis from rats with ages of 1, 2, 3 and 12 months, respectively. Statistical analysis (B) revealed significant decreases in 12 months group compared to 1 and 2 months group. LOX: lysyl oxidase. Fig. S3 ICP graphs and ICP/MAP for rats in different groups and at stages of 1 (A) and 4 (B) weeks after IP. Fig. S4 Sirius red staining (20×) of corpus cavernosum by polarizing microscopy at stages of 1 (A) and 4 (B) weeks. Fig. S5 Transmission electron microscopy of corpus cavernosum (12,000×) at stages of 1 (A) and 4 (B) weeks. Fig. S6 Transmission electron microscopy of collagen in corpus cavernosum (15,000×) at stages of 1 (A) and 4 (B) weeks. [file JCMM-22-2018-s001.doc]

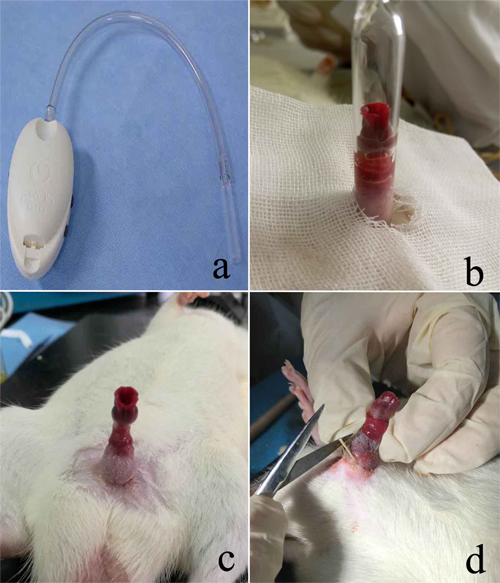


**Fig. S1** The establishment of ischemic priapism (IP) model.


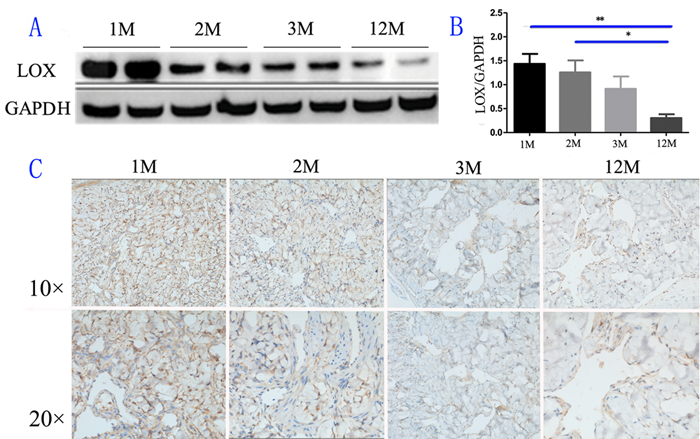


**Fig. S2** Western Blot (A) and immunohistochemistry (IHC) (C) of LOX in penis from rats with ages of 1, 2, 3 and 12 months, respectively. Statistical analysis (B) revealed significant decreases in 12 months group compared to 1 and 2 months group. LOX: lysyl oxidase.


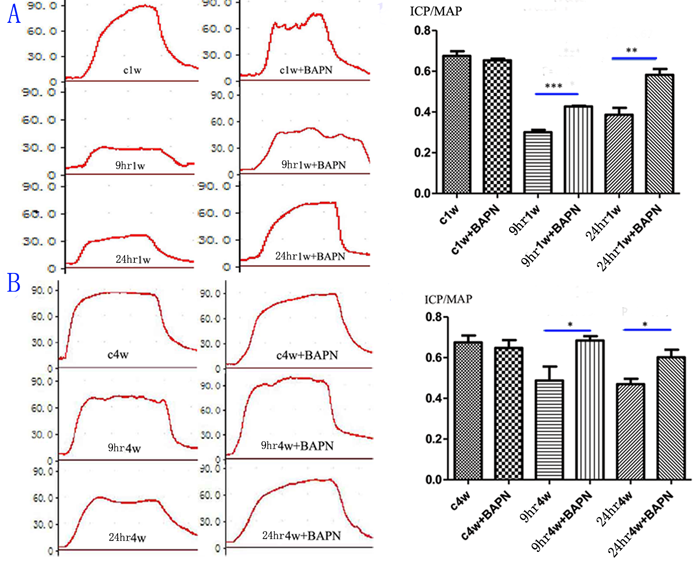


**Fig. S3** ICP graphs and ICP/MAP for rats in different groups and at stages of 1 (A) and 4 (B) weeks after IP.

**
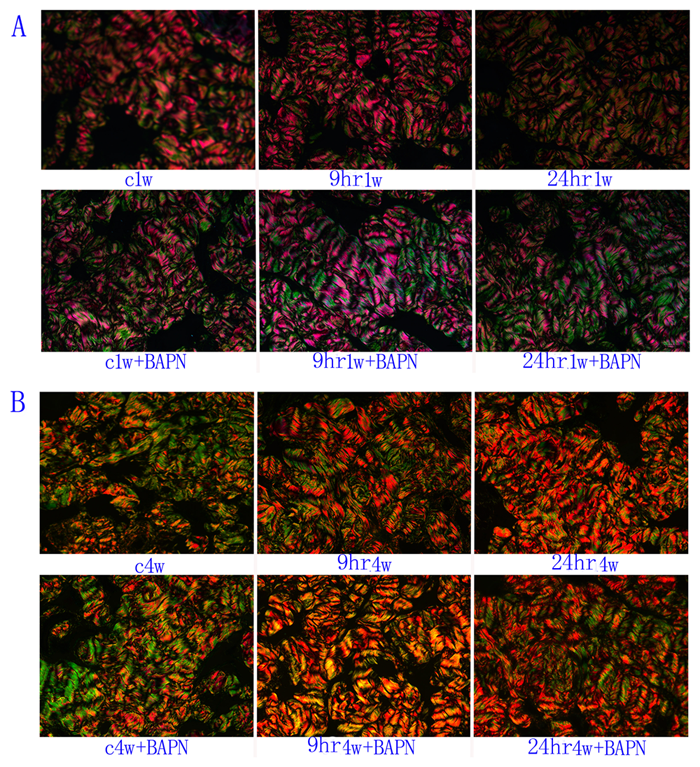
**

**Fig. S4** Sirius red staining (20×) of corpus cavernosum by polarizing microscopy at stages of 1 (A) and 4 (B) weeks.


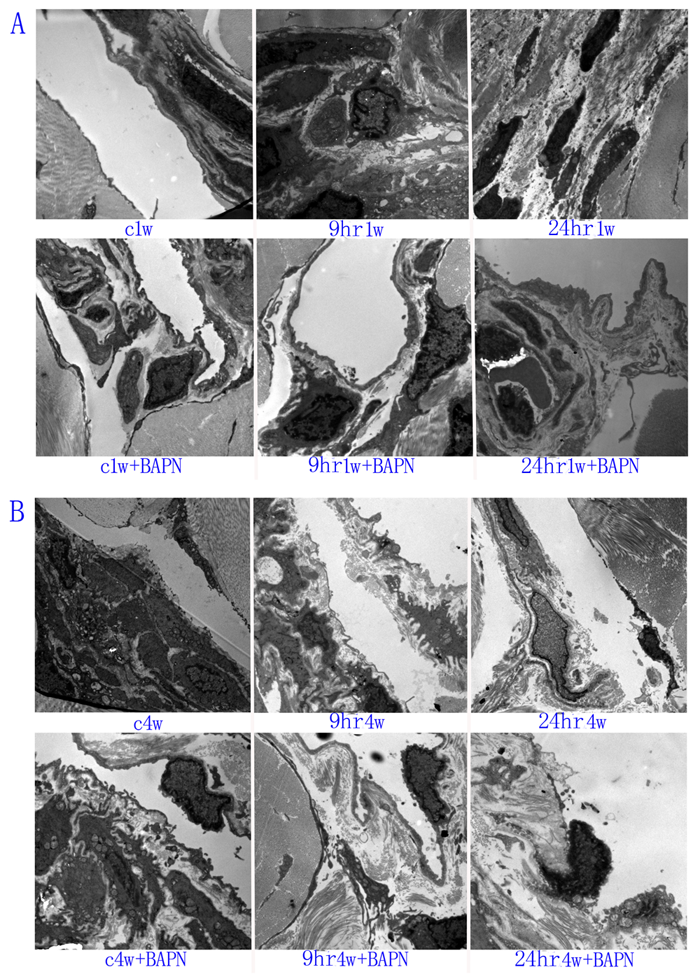


**Fig. S5** Transmission electron microscopy of corpus cavernosum (12,000×) at stages of 1 (a) and 4 (b) weeks.


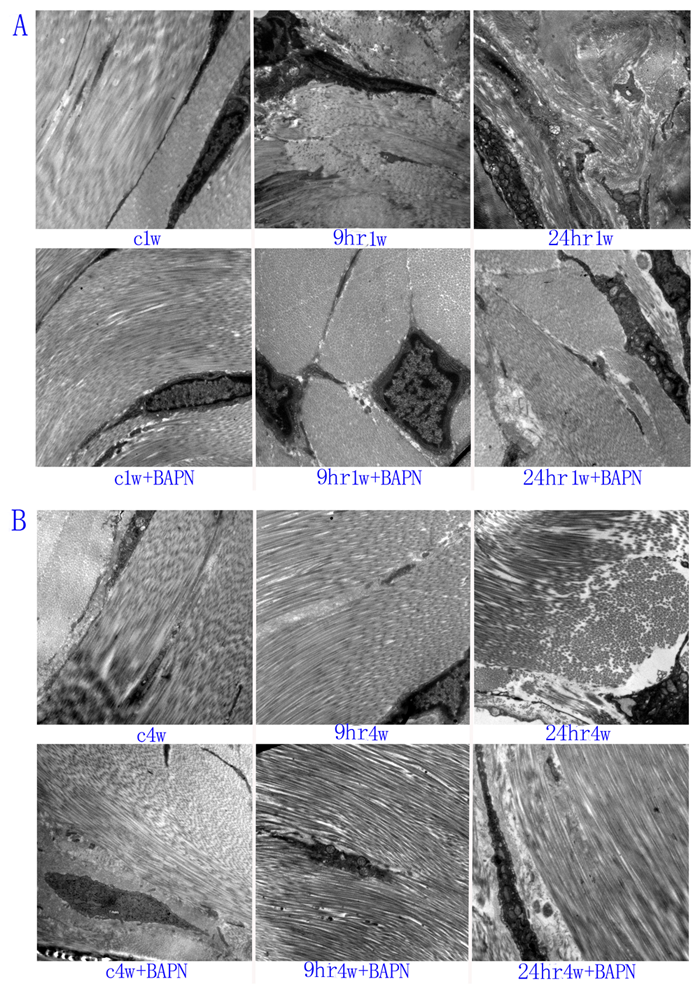


**Fig. S6** Transmission electron microscopy of collagen in corpus cavernosum (15,000×) at stages of 1 (a) and 4 (b) weeks.
